# Terbium- and samarium-doped Li_2_ZrO_3_ perovskite materials as efficient and stable electrocatalysts for alkaline hydrogen evolution reactions

**DOI:** 10.1007/s11356-024-34846-x

**Published:** 2024-08-31

**Authors:** Gobeng R. Monama, Morongwa E. Ramoroka, Kabelo E. Ramohlola, Marema W. Seleka, Emmanuel I. Iwuoha, Kwena D. Modibane

**Affiliations:** 1 SensorLab (University of the Western Cape Sensor Laboratories), 4Th Floor Chemical Sciences Building, University of the Western Cape, Bellville 7535, Cape Town, South Africa; 2 Nanotechnology Research Lab, Department of Chemistry, School of Physical and Mineral Sciences, University of Limpopo (Turfloop), Polokwane, 0727 Sovenga South Africa; 3 DSI-NRF SARChI Chair in Photoelectrocatalytic Hydrogen Production, Department of Chemistry, School of Physical and Mineral Sciences, University of Limpopo (Turfloop), Polokwane, 0727 Sovenga South Africa

**Keywords:** Electrocatalyst, Electrochemistry, Perovskite oxides, Hydrogen evolution reaction, Hydrogen production rate, Tafel mechanism

## Abstract

The preparation of highly active, rare earth, non-platinum-based catalysts for hydrogen evolution reactions (HER) in alkaline solutions would be useful in realizing green hydrogen production technology. Perovskite oxides are generally regarded as low-active HER catalysts, owing to their unsuitable hydrogen adsorption and water dissociation. In this article, we report on the synthesis of Li_2_ZrO_3_ perovskites substituted with samarium and terbium cations at A-sites for the HER. LSmZrO_3_ (LSmZO) and LTbZrO_3_ (LTbZO) perovskite oxides are more affordable materials, starting materials in abundance, environmentally friendly due to reduced usage of precious metal and moreover have potential for several sustainable synthesis methods compared to commercial Pt/C. The surface and elemental composition of the prepared materials have been confirmed by X-ray photoelectron spectroscopy (XPS). The morphology and composition analyses of the LSmZO and LTbZO catalysts showed spherical and regular particles, respectively. The electrochemical measurements were used to study the catalytic performance of the prepared catalyst for hydrogen evolution reactions in an alkaline solution. LTbZO generated 2.52 mmol/g/h hydrogen, whereas LSmZO produced 3.34 mmol/g/h hydrogen using chronoamperometry. This was supported by the fact that the HER electrocatalysts exhibited a Tafel slope of less than 120 mV/dec in a 1.0 M alkaline solution. A current density of 10 mA/cm^2^ is achieved at a potential of less than 505 mV. The hydrogen production rate of LTbZO was only 58.55%, whereas LSmZO had a higher Faradaic efficiency of 97.65%. The *EIS* results demonstrated that HER was highly beneficial to both electrocatalysts due to the relatively small charge transfer resistance and higher capacitance values.

## Introduction

Fossil fuels are becoming distinct and would not last even for the probable future as the principal energy source in the universe (Leggett and Ball 2012; Fulkerson et al. 1990; Arutyunov and Lisichkin 2017; Dincer and Acar 2015). Researchers have been trying to come up with different fuels that are both maintainable and green. Hydrogen has been identified as a potential source of energy that is not depleting and can meet the world’s needs. H_2_ gas has an exceptionally high energy density of nearly 140 MJ/kg, and when it is combusted, it only yields water as a by-product, making it very clean (Arutyunov and Lisichkin 2017; Dincer and Acar 2015; Kim et al. 2014). Hydrogen is the most plentiful element in the world, although it is not freely available. To obtain hydrogen in a useful form, it must be produced by some other means and stored so that it can be used successfully as and when needed (Kim et al. 2014; Osman et al. 2022; Yu et al. 2021).

Hydrogen can be identified as a fuel by colour codes. The colour codes of hydrogen refer to the source or process used to make it. The most prominent hydrogen gases are the high greenhouse-emitting black, brown, and grey hydrogens, which are produced from thermochemical processes such as coal gasification and steam reforming processes, respectively (Osman et al. 2022; Yu et al. 2021). Furthermore, during steam reforming, the produced CO_2_ can be captured to yield blue hydrogen. The purest and most ideal hydrogen is green hydrogen, which is produced from water electrolysis and has zero carbon emissions (Kim et al. 2014; Osman et al. 2022; Yu et al. 2021; Panchenko et al. 2023). Thus, the electrochemical reduction of water at ambient temperature to molecular hydrogen offers a substantial promise for supplying hydrogen (Karchiyappan 2019; Phuruangrat et al. 2009). However, the hydrogen evolution reaction (HER, 2H^+^  + 2e^−^  → H_2_) regularly needs advanced electrocatalysts to increase efficiency and reduce energy costs by reducing overpotential and enhancing HER activity in both acidic and basic solutions (Karchiyappan 2019; Phuruangrat et al. 2009; Guan et al. 2019; Xu and Zhang 2019). Although HER in an acidic condition has a lower overpotential than in a basic environment (Xu and Zhang 2019), there is still a promise in the basic medium since non-precious metal-based catalysts can be employed (Xu and Zhang 2019; Safizadeh et al. 2015; Shen et al. 2020). Platinum (Pt) and its alloys are still by far the benchmark electrocatalysts for hydrogen evolution, regardless of whether the reaction is in an acidic or basic medium (Yang et al. 2022; Dubouis et al. 2018). The widespread use of Pt and its alloys for H_2_ production is constrained by their high cost and scarcity. As a result, great efforts must be made to develop low-cost alternative catalysts that are durable, non-precious metal and efficient for HER in their basic condition (Yang et al. 2022; Dubouis et al. 2018; Lu et al. 2019).

Various noble-metal-free electrocatalysts such as transition metal carbides, sulphides, phosphides and carbon-based electrocatalysts have been recognised as potential electrocatalysts for HER; regrettably, most of these catalysts show poor intrinsic activity and stability in base solutions (Dubouis et al. 2018; Lu et al. 2019; Hughes et al. 2021). Lately, inorganic lead-free metal perovskites have lately received continual attention because of their non-toxicity, low-cost, as well as excellent stability and electrochemical properties (Zhao et al. 2023; Li et al. 2022; Wang et al. 2023; Mo et al. 2021). Perovskite oxides with a common formula of ABO_3_ have gained significant attention in HER because of their low cost, low toxicity and rich redox chemistry (Dou et al. 2021; Yang et al. 2023; Wu et al. 2023). Additionally, their A site, which is usually alkali earth elements or lanthanides, and transition metals on the B site can easily be tailored for specific catalytic applications (Yang et al. 2023; Wu et al. 2023) Nevertheless, perovskite oxides do not meet the activities of platinum group metal HER electrocatalysts, but they show greater activity towards counterpart reactions such as oxygen reduction (ORR) and oxygen evolution (OER) reactions (Alom et al. 2022; Dou et al. 2021; Yang et al. 2023; Wu et al. 2023). This is due to their ineffective conversion of hydrogen intermediates to H_2_ for oxides, particularly in alkaline HER (Guan et al. 2019).

Several approaches have been recommended to optimise the HER properties of perovskites through doping, defect engineering and morphology control since electrocatalytic reactions commonly occur on the surface of the electrocatalyst (Wu et al. 2023; Si et al. 2022). Among several approaches, partial substitution of the A-site or B-site of the perovskites has been identified as a preferred way to fine-tune their structure from pristine ABO_3_ into double perovskites with the general formula A_x_A′_1−x_B_x_B′_1−x_O_3_ (Aziz et al. 2023). Furthermore, replacing the A-site cation can lead to improvements in electrical conductivity, Goldschmidt tolerance factor and A-site order reactions (Alom et al. 2022). For example, Wu et al. (2023) recently employed lanthanide metals to dope RBaCo_2_-O_5.5_ + *α* (*α* = 1/4) to form oxygen vacancies (*V*_*o*_) and high-valence Co^4+^ in perovskite oxide, which resulted in effective water dissociation and the ability to release hydrogen swiftly. They further achieved a better performance of the doped electrocatalyst with an overpotential reduction of over 200 mV than that of the undoped electrocatalyst at 0.36 mA/cm^2^ and a low Tafel slope of 91 mV/dec. In another study, Dou et al. (2021) reported LnBaCo_2_O_5 + δ_ as one of the best-performing non-precious metal-based perovskite catalysts in alkaline media, with an overpotential of 156 mV at 10 mA/cm^2^ and a low Tafel slope of 64.4 mV/dec. Furthermore, doping with lanthanides such as terbium (Tb) and samarium (Sm) can induce oxygen vacancies onto the structure of the catalysts and enhance the HER (Ahmad et al. 2023). Synergistic effect of doping-induced oxygen vacancies, in-built Tb^4+^/Tb^3+^ redox centres and heterojunction on the photocatalytic activity of Sm-doped ZnO/Y-doped Tb_2_O_3_ for H_2_ evolution (Ahmad et al. 2023). Ahmad and coworkers (2023) demonstrated the photocatalytic effect of HER on the oxygen vacancies induced by doping Tb and Sm. The enhanced HER production was attributed to the generation of oxygen vacancies, which resulted in improved optical response, spatial separation, quick charge carrier transport and a high density of catalytic sites that improved the performance of H_2_ evolution. According to the literature, there are no reports of substituting the A-site of the perovskite for electrocatalytic HER.

In this work, we report for the first time on the synthesis and characterizations of LiSmZrO_3_ (LSmZO) and LiTbZrO_3_ (LTbZO) perovskite oxide as an active and robust electrocatalyst for HER in a basic medium. Metal^3+^ ions have been reported to play a significant role in increasing the lattice’s oxygen vacancy and have yielded favourable results in recent studies (Si et al. 2022). Samarium and terbium were doped in the perovskite structure at the A-site to determine their roles in hydrogen production. Through precise control of the doping amount of M^3+^, LTbZO and LSmZO materials showed good HER activities in 1.0 M KOH with an overpotential of less than 505 mV to reach a current density of 10 mA/cm^2^. This study provides a feasible method for developing highly effective lanthanide-based perovskites using A-site doping for the alkaline HER.

## Experimental

### Materials

Chemical reagents, lithium carbonate (Li_2_CO_3_, 99.9%), zirconium carbonate (ZrO_2_, 99%), citric acid (C_6_H_8_O_7_, 99.9%), terbium nitrate hexahydrate (Tb(NO_3_)_3_6H_2_O, 99.99%), samarium nitrate hexahydrate (Sm(NO_3_)_3_6H_2_O, 99.99%), platinum carbon (Pt/C), NMP (C_5_H_9_NO), PVDF (C_2_H_2_F_2_)_n_ − and nitric acid (HNO_3_, 55%) were obtained from commercial sources and used without further purification.

### Synthesis of an electrocatalyst

**Scheme 1:**
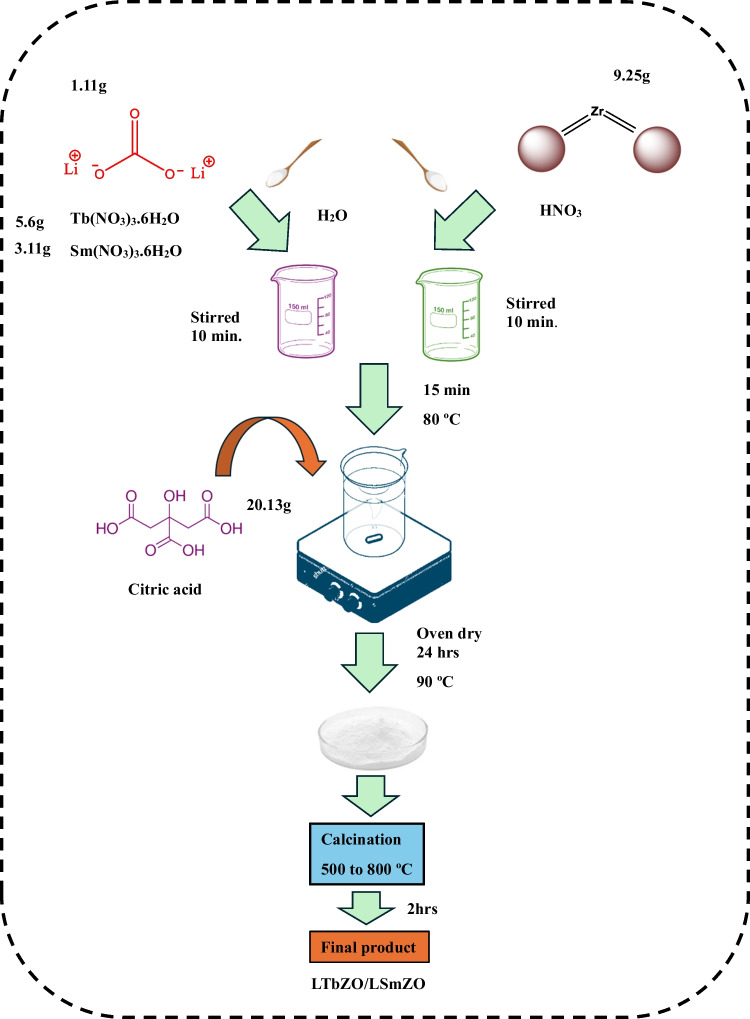
Synthesis of LTbZO and LSmZO

The LTbZO and LSmZO materials (Scheme 1) were prepared through sol–gel method (Sarmad et al. 2022). First, 1.11 g of Li_2_CO_3,_ 5.6 g Tb(NO_3_)_3_6H_2_O or 3.11 g Sm(NO_3_)_3_6H_2_O were mixed in deionized water and on separate beaker 9.25 g ZrO_2_ was dissolved in dilute HNO_3._ The solutions were stirred for 10 min in separate beakers to uniformly disperse the solutes. The two solutions were mixed and stirred for further 15 min. Furthermore, mixed solution was stirred on hot plate at a temperature of 80 °C for few hours, and 20.13 g of citric acid was poured slowly into the solution to complete the formation of a gel. Then, the gel was transferred in an oven at 90 °C overnight to dry. The as-synthesized sample was calcined at 300–900 °C for 2 h to remove organic substances.

### Characterization techniques

Several analytical techniques were used to characterise the catalyst samples. The technique employed for the investigation of phase structure was an X-ray diffractometer (XRD), which was used to investigate phase structure at 35 kV and 40 mA with Cu-Ka radiation (*k* = 0.154 nm). Diffractograms were obtained using a Malvern Panalytical Aeris diffractometer with a PIXcel detector and fixed slits with Fe-filtered Co-Kα radiation. The phases were identified using X’Pert Highscore Plus software, as well as the PAN-ICSD and ICDD PDF-4 databases. The formation and characterization peaks of LZO, LSmZO and LTbZO were analysed by Fourier-transform infrared spectroscopy (FT-IR). The thermal stability of the catalyst was analysed by a thermogravimetric analyzer (STA) from room temperature to 1000 °C with a heating rate of 10 °C/min in the air. Scanning electron microscopy (SEM) and the high-resolution transmission electron microscope (HRTEM) were used to observe the structure and morphology of the catalysts. X-ray photoelectron spectroscopy (XPS) was acquired using Shimadzu Axis Supra + with a fresh fracture surface that was also etched by 4 kV argon-ion for 30 s in situ in the instrument. The XPS spectra were calibrated based on the binding energy of C1s (284.8 eV).

### Electrochemical measurement

Electrochemical measurements were performed using a three-electrode system on electrochemical workstations on a biologic instrument (VMP300, EC-Lab software). The working electrode was made of bare or modified nickel foam. Platinum wire and Ag/AgCl (saturated with KCl) were used as counter and reference electrodes, respectively. Firstly, the nickel foam substrate was cut into 0.9 cm × 0.7 cm and treated with 3 M HCl, distilled water and ethanol, respectively, for 15 min independently. These pieces of nickel foam were dried at 60 °C for 2 h. The slurry was prepared by mixing 15 mg of active material, 3 mg of carbon black and 2 mg of PVDF (–(C2H2F2)n–) in 100 μl of NMP (C_5_H_9_NO). To ensure thorough dispersion, the solution was sonicated for 60 min. Finally, the slurry was drop-cast on the nickel foam and left to dry overnight. Then, CV scans were performed at different scan rates of 20 mV·s^−1^ to 100 mV·s^−1^ between 0.00 and 0.70 V vs. Ag/AgCl until the CV curves were reproducible. Subsequently, linear sweep voltammetry (LSV) at a scan rate of 10 mV/s was conducted. Electrochemical impedance spectra (*EIS*) were collected at a frequency of 10 kHz to 0.1 Hz at an amplitude of 5 mV. In a 0.1 M KOH solution, these techniques were used to study the catalytic performance of the prepared catalysts.

## Results and discussion

### Structural characterization

**Fig. 1:**
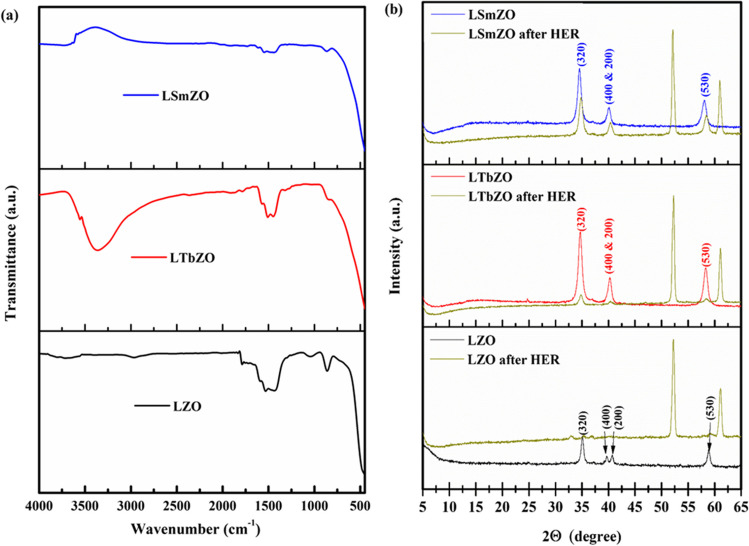
**a** FTIR spectra and **b** XRD patterns of LZO, LTbZO and LSmZO

The FTIR spectra of the synthesised materials LZO, LTbZO and LSmZO are recorded in the range of 4000–500 and 500 cm^−1^, respectively, as shown in Fig. 1a. The two broad absorption and desorption bands in the 3496–3250 cm^−1^ range are due to the stretching vibrations of the adsorbed water (Yakout and Hassan 2014; Rosid et al. 2019). The peaks observed at 1529.62 and 1448.6 cm^−1^ are connected to the Zr-O stretching mode. The peaks around 800 cm^−1^ are due to the bending vibration of hydroxyl groups bound to zirconium oxide (Yakout and Hassan 2014; Onghena et al. 2018). Both LSmZO and LTbZO materials observed similar bands that shifted towards lower wavenumbers with the presence of lanthanide (Tb^3+^ and Sm^3+^) when compared to LZO material without lanthanide (Onghena et al. 2018). Crystallinity is a critical factor in determining the overall performance of any material synthesised for use in a variety of energy applications. Sol–gel-synthesised materials LSmZO and LTbZO perovskites are checked for crystallinity as well as calculating crystallite sizes shown in Fig. 1b. LTbZO and LSmZO showed four sharp diffraction peaks at 2*θ* = 34°, 41°, 58° and 70° which can be attributed to the crystalline nature of LZO, and this is consistent with our previous works (Xue et al. 2020; Natalia et al. 2018). This result was further supported by the determination of crystallite size for both samples using the Scherrer formula. (Jaffri et al. 2023).

**Fig. 2:**
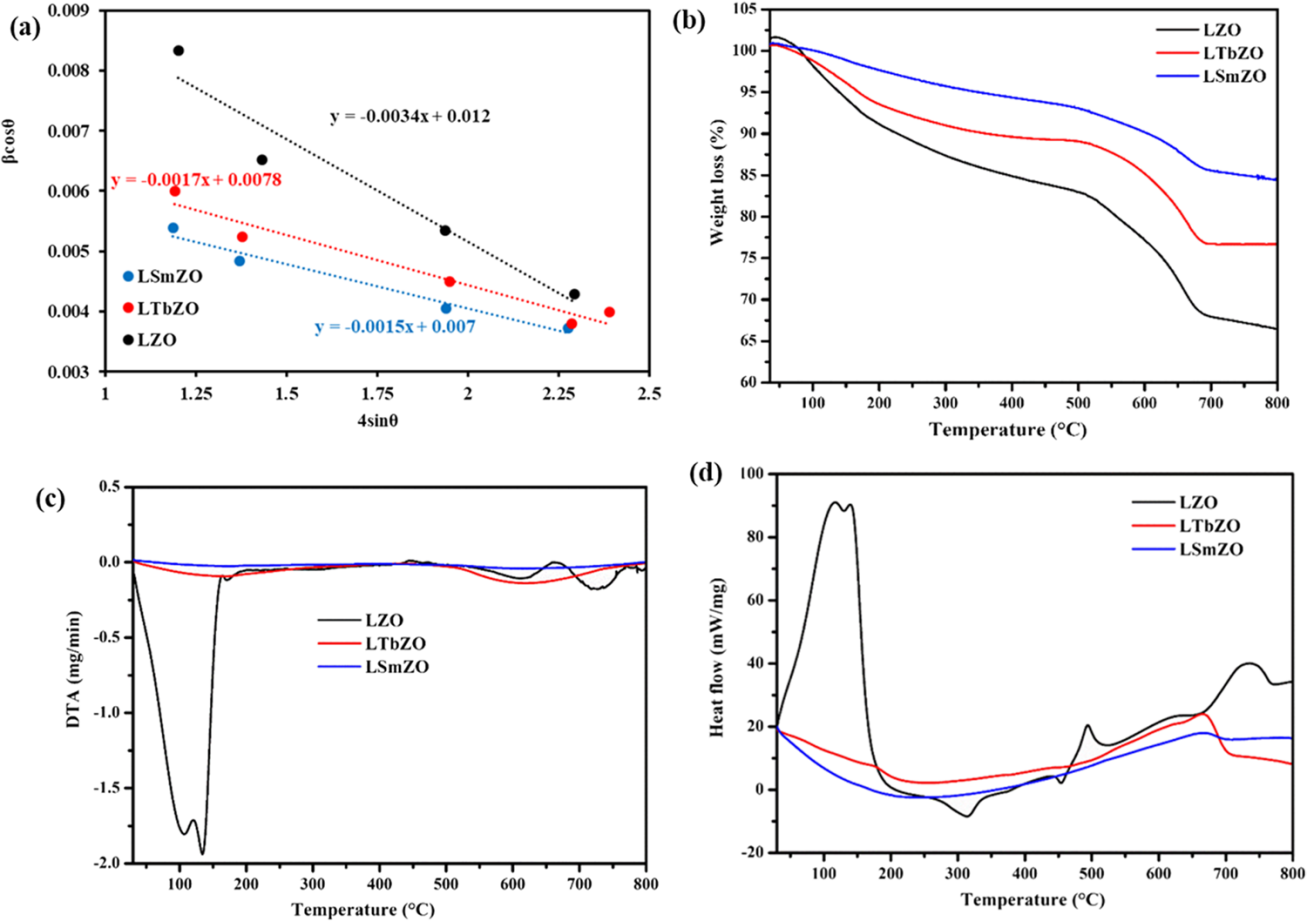
**a** Williamson–Hall plot, **b** TGA curves, **c** DTA and **d** DSC curves of LZO, LTbZO and LSmZO

The symbol *β* signifies the full-width at half-maximum (FWHM) in radians; *λ* stands for the 1.5406 Å wavelength of Cu-Kα radiation; and *θ* is the Bragg’s angle. The structural parameters of the synthesised materials are calculated and listed in Table 1. The observed crystallite size was due to the lattice strain in the materials. As a result, the strain (*ε*) and dislocation density (*ρ*) of the LZO, LSmZO and LTbZO are computed according to Maponya et al. (2023) and presented in Table 1. The strain (*ε*) values were found 1.56 × 10^−3^, 1.46 × 10^−3^ and 1.57 × 10^−3^ for LZO, LSmZO and LTbZO, respectively. The *ρ* values were determined to be 20.30 × 10^14^ lines/m^2^ for LZO, 17.71 × 10^14^ lines/m^2^ for LSmZO and 20.38 × 10^14^ lines/m^2^ for LTbZO. It showed that the introduction of Sm has impact on the *ε* and *ρ* values. The smaller *ρ* value of LSmZO indicates that the Sm containing material has a higher degree of crystallinity as compared to LZO and LTbZO. In addition, the crystallite size (*D*) and strain (*ε*) values were also determined using Williamson–Hall (W–H) plot according to *β*cos*θ* = 0.9*λ*/*D* × 4* ε*sin*θ*. The plot as given in Fig. 2(a) shows a relationship of *β*cos*θ* as a function of 4sin*θ*. The *D* values were obtained from the *y*-intercept, whereas the strain values were obtained from the slope of the graph. When the particle size becomes smaller, more lattice strain can be expected in the nanoparticles resulting in the broadening in the XRD peaks. Therefore, W–H plot gives a more accurate particle size estimation. The grain size obtained from the W–H plot for the LSmZO was 19.81 nm, and LTbZO was 17.78 nm as compared to 11.55 nm of LZO. The trend followed by the grain sizes obtained from W–H plot was similar as the crystallite sizes calculated by the Debye–Scherrer formula. The negative slope of the fitted lines in the W–H plot shows the presence of compressive strain in the lattice of LSmZO and LTbZO.

**Table 1:** Structural parameters obtained from XRD

Materials	2-theta (degree)	d-spacing (nm)	FHWM	D (nm)	Strain	Dislocation density (10^14^ m^−^)
LZO	34.87	2.53	6.50 × 10^–3^	22.20	1.56 × 10^–3^	20.30
LSmZO	34.58	2.55	6.11 × 10^–3^	23.76	1.46 × 10^–3^	17.71
LTbZO	34.71	2.54	6.56 × 10^–3^	22.15	1.57 × 10^–3^	20.38

### Thermal properties

The thermal properties of LZO, LTbZO and LSmZO were investigated using TGA and DSC. Figure 2 depicts TG thermograms (a) and their corresponding weight loss derivatives (b). It is noteworthy that LZO, LTbZO and LSmZO exhibited two degradation steps, while LTbZO and LSmZO showed improved thermal stability, indicating the chemical changes in the structure and their effect on LZO’s thermal stability. All samples showed a small dehydration step up to 200 °C, constituting less than 10.0 wt%, 6.5 wt%, and about 2.0 wt% of the LZO, LTbZO and LSmZO, which was attributed to the release of absorbed solvent, respectively. This degradation feature was more pronounced in LZO (Fig. 2(b)). The temperature rise resulted in a clear exothermic peak at 317 °C and a subsequent weight loss, both of which were caused by the thermal degradation of LZO, while LTbZO and LSmZO did not show an exothermic peak around 317 °C (Fig. 2(c)). The second degradation step for LZO, LTbZO and LSmZO was observed around 510 °C and was attributed to the bound water and organic ligands (Woods et al. 2017; Goda et al. 2019; Otaki et al. 2023). Remarkably, the residual weight losses of 32.5 wt%, 22.5 wt% and 15.0 wt% at a temperature of 800 °C correspond to the final residues of LZO, LTbZO and LSmZO, respectively. The differential scanning calorimetry curves of LZO, LTbZO and LSmZO exhibit thermograms that are comparable to those shown in Fig. 2(a). Furthermore, the exothermal peak at 725 °C might be due to the crystallisation of LZO, while LTbZO and LSmZO exhibited a small exothermic reaction peak at 650 °C, and this transition indicates the crystallisation of the materials (Fig. 2(c)).

### Morphological and XPS characterization

**Fig. 3:**
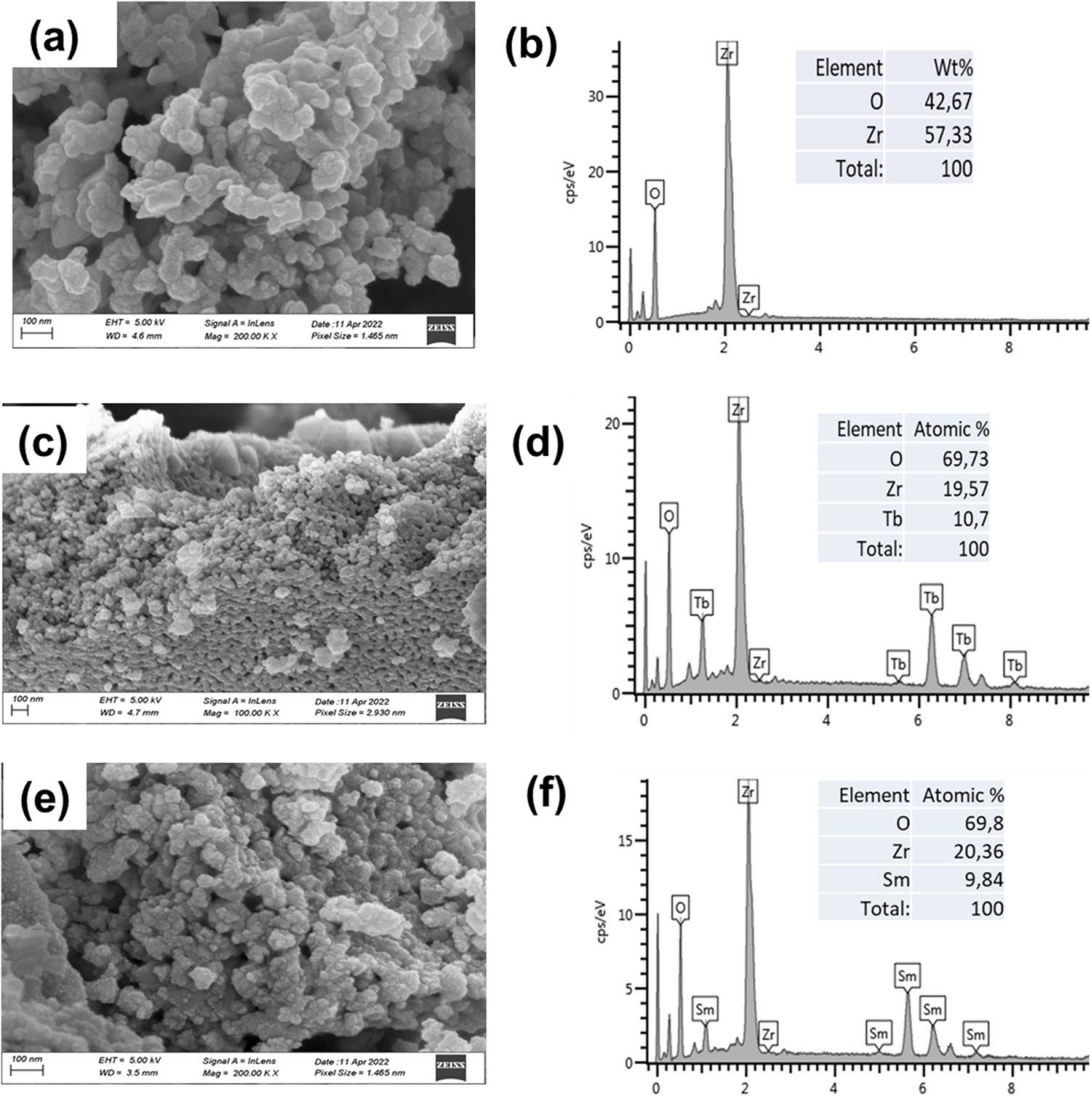
**a** SEM image of LZO, **b** EDS showing chemical composition of LZO, **c** SEM image of LTbZO, **d** EDS showing chemical composition of LTbZO, **e** SEM image of LSmZO and **f** EDS showing chemical composition of LSmZO

The morphological properties of the prepared LTbZO and LSmZO samples were investigated. Figure 3 shows the SEM and EDS images of these samples. The SEM image (Fig. 3a) of the as-prepared LTbZO crystalline gel demonstrated solid, agglomerated and regular particles. The EDS analysis is done in order to deduce the elemental composition of the prepared materials, and the results are shown in Fig. 3b. All the expected elements, Tb, O and Zr, were present, with the exception of the Li element, which has low absorption energy and, as such, is undetectable in the EDS (Hodoroaba 2020). The crystal morphologies of LSmZO are demonstrated in Fig. 3c. The synthesised sample shows rough, spherical particles with an average diameter of ~ 50 nm. This is consistent with the prepared material’s XRD patterns, which were crystalline for LSmZO. According to the corresponding EDS results (Fig. 3d), the observed elements are Sm, Zr and O, which confirms the successful synthesis of the catalyst.

**Fig. 4:**
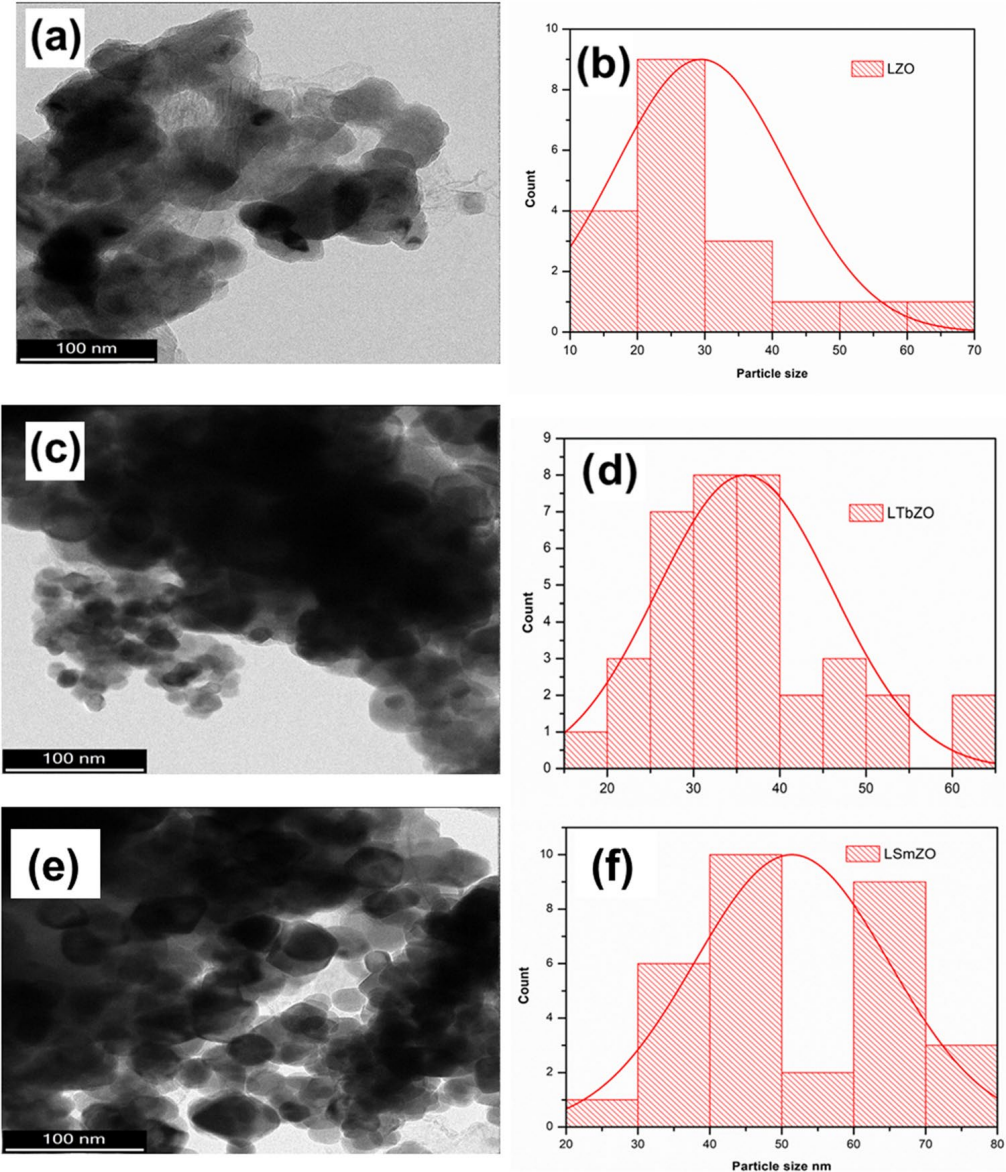
**a** TEM image of LZO, **b** particle size distribution of LTbZO, **c** TEM image of LTbZO, **d** particle size distribution of LTbZO, **e** TEM image of LSmZO and **f** particle size distribution of LSmZO

Figure 4 shows the TEM images and particle size distribution of LTbZO and LSmZO obtained from the Gaustic plot. Figure 4 a shows that the TEM image of LTbZO confirms crystalline and spherical morphology. As confirmed by the TEM observation in Fig. 4a, the size of the nanoparticles is 30–40 nm with some agglomerates. Agglomeration is caused by nanoparticle sintering during calcination (Guo et al. 2017). The detailed crystal morphologies of LSmZO material are evaluated by TEM, as shown in images in Fig. 4b. LSmZO particles show a clear, nanospherical and smooth surface. The mean particle size for LTbZO and LSmZO perovskites ranges from ∼30–45 nm. There was a slight decrease in the particle size of LTbZO (averaging 35 nm) as compared to that of LSmZO perovskite (45 nm). The small mean particle size of LTbZO is consistent with the high *ECSA* (given below), and the results will enhance the material’s HER activity.

**Fig. 5:**
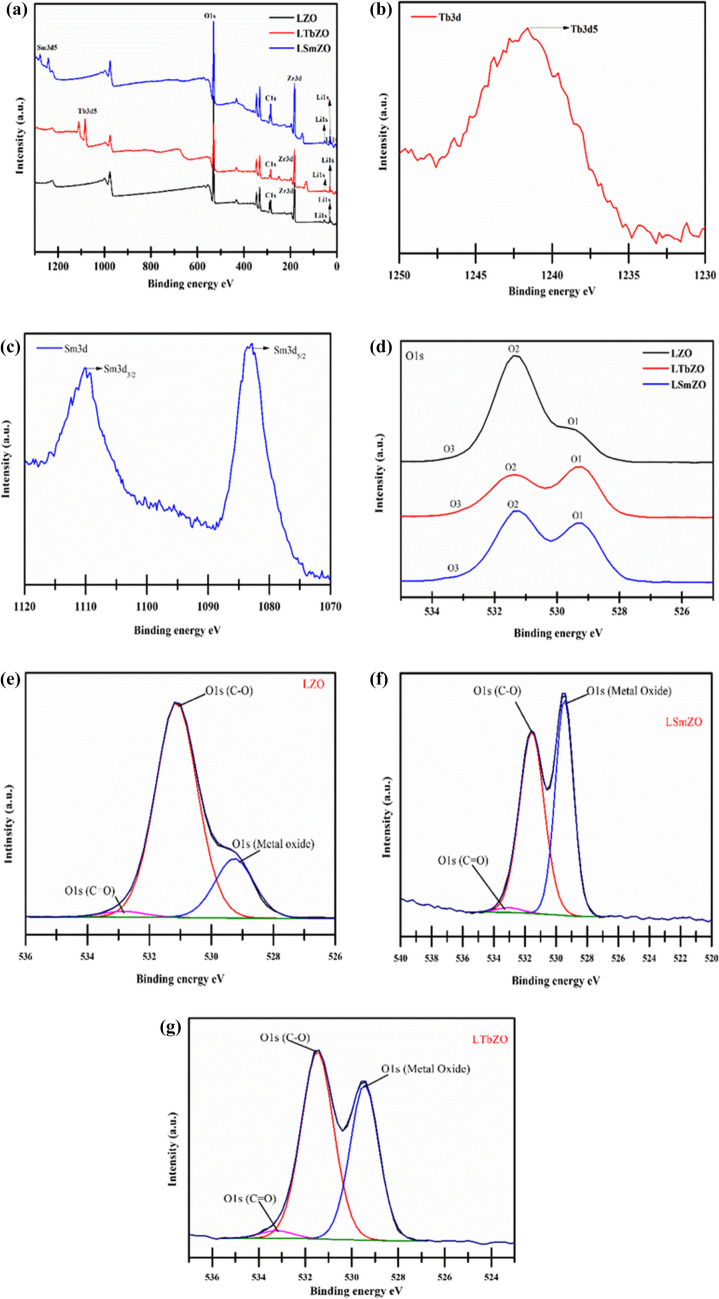
**a** Survey scan spectra, **b** Tb3d spectrum of LTbZO, **c** Sm3d spectra of LSmZO, **d** O 1 s of LZO, LTbZO and LSmZO, **e** deconvulotion of O1s LZO, **f** LSmZO O1s and **g** O1s of LTbZO

X-ray photoelectron spectroscopy (XPS) was carried out to confirm the surface elemental composition of the LSmZO and LTbZO samples. A full survey scanning spectrum in Fig. 5a verifies the existence of Li, Sm, Tb, Zr and O elements in both our samples, which is in agreement with the EDS results. In the Tb3d spectrum (Fig. 5(b)), the peak at 1241.2 eV corresponds to Tb 3d_5/2_, which also indicates that terbium is in the Tb^3+^ oxidation state (Huh et al. 2021). In Fig. 5(c), which shows the high-resolution Sm 3d spectrum, the peaks located at 1114.1 and 1082.9 eV are attributed to Sm 3d_3/2_ and Sm 3d_5/2_, respectively, with an energy difference of 32.0 eV, indicating the existence of Sm^3+^ (Mariyappan et al. 2020; Muscas et al. 2022). The O 1 s XPS spectra of the prepared samples are shown in Fig. 5d, which gives an analysis of the catalyst’s surface as to which species of oxygen are present. The deconvolution shows three notable oxygen peaks. The lowest binding energy peak may be assigned to lattice O1 species from lanthanide elements (529.6 eV) (Chang et al. 2020; Richards et al. 2022). O2 can be assigned to surface hydroxyl species (OH −) at 531.5 eV (Richards et al. 2022), and O3 around 533.4 eV is typically ascribed to water (Chang et al. 2020; Richards et al. 2022). The reduction of area and intensity of the O2 species in the LSmZO and LTbZO, as shown in Fig. 5d, confirms defects in the form of oxygen vacancies (Richards et al. 2022). The surface chemistry and properties of the materials are studied by deconvolution of O1s, as shown in Fig. 5(e–g). The LSmZO lattice oxygen (metal oxide) had higher intensity than the rest of the materials which suggests good crystallinity, and these results are supported by XRD, whereby smaller dislocation density (*ρ*) value was observed (Whitten et al. 2024). The results in all materials (Fig. 5e–g) show significant intensity of surface hydroxyl (C-O) which is known to be good for catalytic activity and also has impact on adsorption capacity of the catalyst (Merino et al. 2006). Finally, there is a small amount of adsorbed water in all our reported materials. This low C = O percentage observed suggests minimal surface contamination, which is beneficial to our materials catalytic activity (Whitten et al. 2024; Merino et al. 2006).

### Electrochemical properties

#### Cyclic voltammetric studies

**Fig. 6:**
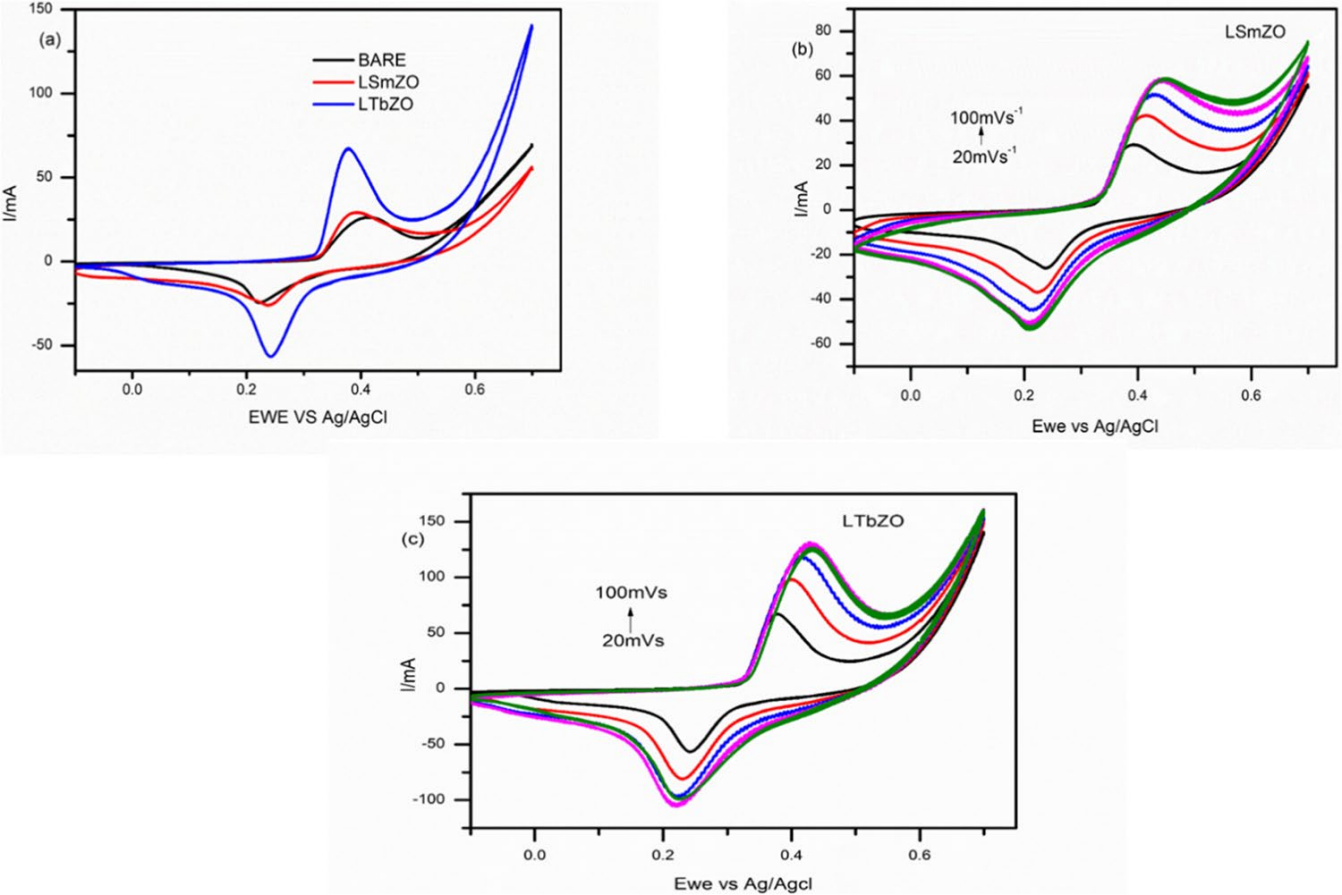
**a** CV curves of bare, LSmZO and LTbZO at 20 mV·s^−1^ in 1.0 M KOH. **b** CV curves at different scan rates for LSmZO from 20 to 100 mV·s^−1^ in 1.0 M KOH. **c** CV curves at different scan rates for LTbZO from 20 to 100 mV/s in 1.0 M KOH

The electrochemical characterization of the nickel foam (bare), LSmZO and LTbZO were performed using cyclic voltammetry in 1.0 M KOH at a scan rate of 20 mV·s^−1^. Cyclic voltammograms are acquired for bare, LSmZO and LTbZO, as shown in Fig. 6(a). Cyclic voltammogram of bare and LSmZO showed almost similar superficial redox peaks, while LTbZO showed the increased cathodic and anodic peaks around 0.22 V and 0.37 V, respectively. The enhancement in the oxidation and reduction peaks of LTbZO suggests faster electron transfer LTbZO as electrocatalyst. It should be noted for all the reported materials bare, LSmZO and LTbZO, reversible redox peaks were observed.

The cyclic voltammetry was used to calculate the highest occupied molecular orbital (HOMO) and lowest unoccupied molecular orbital (LUMO) (Mikolajczyk et al. 2018). From Table 2, the estimated HOMO energy level values were − 4.55, 5.25 and − 5.20 eV for bare, LSmZO and LTbZO, respectively. The LUMO energy were deduced from the reduction potential following Eq. (3) (Mikolajczyk et al. 2018; Peljo and Girault 2018). From the Table, the calculated LUMO energy level values were − 4.94, − 5.32 and − 4.57 eV for bare, LSmZO and LTbZO, respectively. The good chemical reactivity of the electrocatalyst can be associated with the lower optical band gap as materials with lower band gap possess less stability. The electrochemical band gap (*E*_*g*_) was estimated from the difference between the *E*_*HOMO*_ and *E*_*LUMO*_, and were 0.60 eV (bare), 0.063 eV (LSmZO) and 0.63 eV (LTbZO). The effect of scan rates was determined to further investigate the kinetics of the samples. It is observed in Fig. 6(b) and c), when the scan rate is raised from 20 to 100 mV·s^−1^, the peak current also increases with a shift of potential toward more negative value for cathodic peaks and more positive value in anodic peak. These observations suggest a diffusion-controlled process (Ramohlola et al. 2018). The enhancement of the peak current with an increased scan rate was also due to the thinning of the diffusion layer at a higher scan rate (Van Benschoten 1983).

**Table 2:** Electrochemical parameters of the prepared materials

Electrochemical parameters	Bare	LSmZO	LTbZO
*E*_1/2_^ox^, V	0.4	0.50	0.45
*E*_1/2_^red^, V	0.2	− 0.57	0.18
*E*_*LUMO*_, eV	− 5.15	− 5.32	− 4.57
*E*_*HOMO*_, eV	− 4.55	− 5.25	− 5.20
*E*_*g*_, eV	− 0.6	0.063	− 0.63
*Γ* × 10^−6^ mol.cm^−2^	5.11	4.84	6.22
*D* × 10^−6^ cm^2^·s^−1^	0.347	3.066	5.29
*C*_*dI*_, F·cm^−2^	0.703	0.963	1.169
*ECSA*, cm^2^	322.86	442.25	536.74

**Fig. 7:**
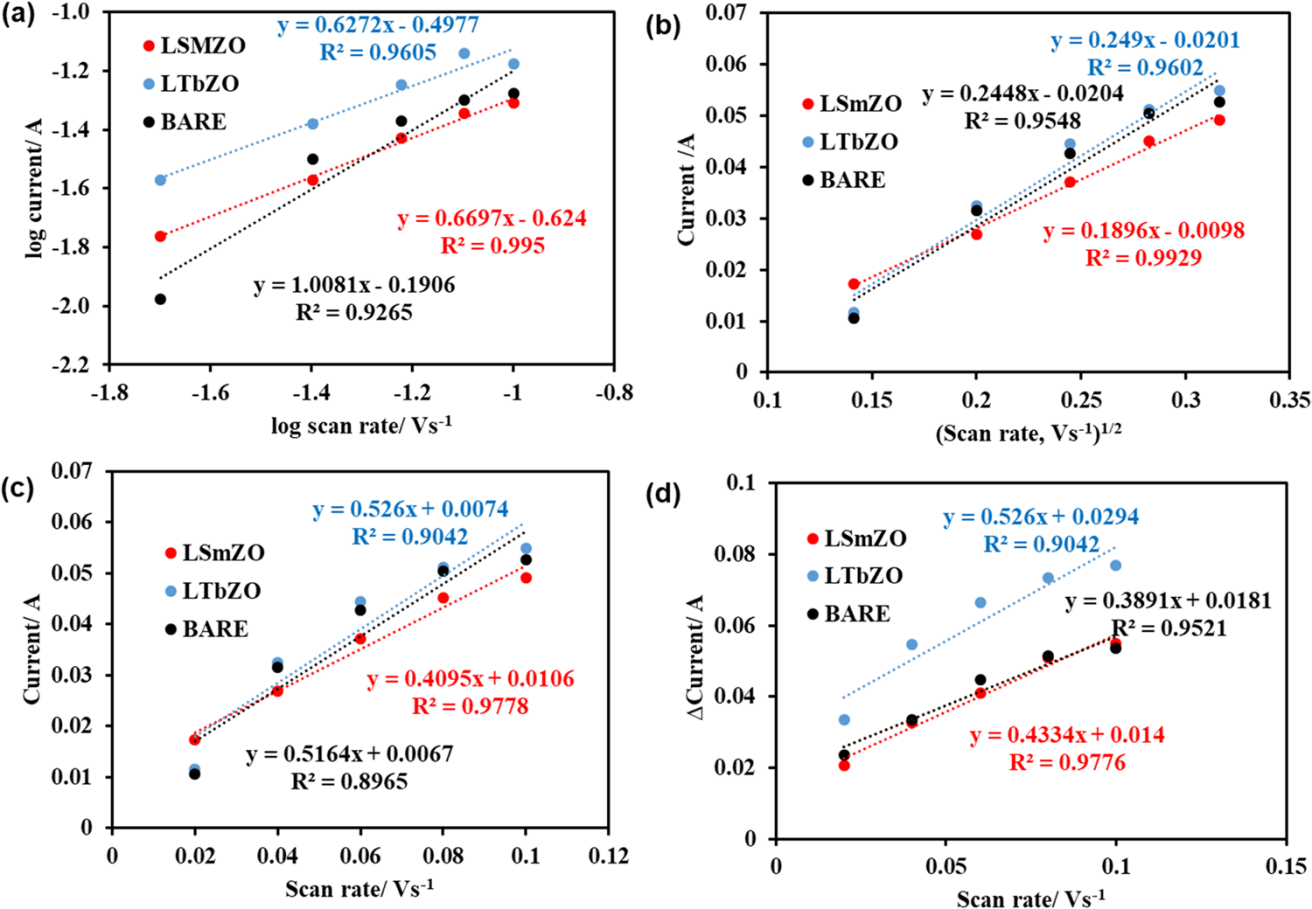
**a** Plot of log of peak current vs. plot of log of scan rate in 1.0 M KOH, **b** plot of peak current against square root of scan rate in 1.0 M KOH, **c** plot of peak current against scan rate in 1.0 M KOH and **d** plot used to estimate *ECSA* for bare, LSmZO and LTbZO in 1 M KOH

The effect of scan rate was used to study the nature of electrode process occurring at the electrode surface of the bare, LTbZO and LSmZO materials. Figure 7(a) shows a plot of logarithm of peak current, log (ip), vs. the logarithm of scan rate, log *ѵ*. In this work, we determined the slope of the fit of log (*i*_*p*_) vs. log *v* which can suggest whether the reaction is controlled by diffusion or adsorption. This relationship was found to be linear with obtained slope of 0.62, 0.66 and 1.00 for LTbZO, LSmZO and bare, respectively. The observed slopes of LTbZO and LSmZO which are near to the theoretical value of 0.5 indicate that process is diffusion controlled (González-Meza et al. 2019; Henstridge et al. 2012). The slope of close to theoretical value of 1 for bare suggests the process dominantly controlled by adsorption (Leftheriotis et al. 2007). On the other hand, the relationship between the square root of the scan rate and the peak current is shown in Fig. 7(b). The plot of peak current vs. square root of scan rate (*ν*^1/2^) forms a good linear relationship and is linear, which indicates that it is a typical diffusion controlled current process (González-Meza et al. 2019; Henstridge et al. 2012; Leftheriotis et al. 2007). To support the observed results, diffusion coefficient (*D*) is calculated using slope from Fig. 7(b).

As summarized in Table 2, the LTbZO has a high value of *D* (5.29 × 10^−6^ cm^2^·s^−1^), LSmZO (3.066° 10^–6^ cm^2^·s^−1^) and bare (0.347 × 10^−6^ cm^2^·s^−1^). This observation means LTbZO will diffuse electrons much faster than the LSmZO and bare which is also compatible with results from Fig. 7(a). To further evaluate the electrochemical behaviour of the bare, LSmZO and LTbZO, the influence of scan rate on average values of both the anodic and cathodic peak currents is analysed, as shown in Fig. 7(c), and the linear relationship is observed. According to literature, the peak current can be correlated with the concentration of electroactive species on the electrode surface (Adam and Newair 2022; Alshammari et al. 2023; Jović 2022; Yamada et al. 2022). The calculated electrode surface coverage concentrations of bare was 5.11 mol·cm^−2^, LSmZO 4.84 mol·cm^−2^ and LTbZO 6.22 mol·cm^−2^. The determination of the electrochemical active surface area (*ECSA*) is vital to study the behaviour of the electrocatalyst. One could assess *ECSA* by determining the capacitance (*C*_*dl*_) in the double-layer region. The value of *C*_*dl*_ can be denoted from plotted current differences vs. scan rate, which signifies the active surface area (Alshammari et al. 2023).

Figure 7(d) shows the CV curves of the bare, LSmZO and the LTbZO electrocatalysts. The plotted change in current vs. scan rate was linearly fitted to calculate the slope value, which was used as *C*_*dl*_ (Alshammari et al. 2023; Jović 2022). The *C*_*dl*_ values were 0.703 F·cm^−2^, 0.963 F·cm^−2^, 1.169 F·cm^−2^ for the bare, LSmZO and the LTbZO, respectively in 1.0 M KOH medium. The calculated *ECS*A values were 322.86 cm^2^, 442.25 cm^2^ and 536.74 cm^2^ for the bare, LSmZO and the LTbZO, respectively. This result revealed that LTbZO is having the supreme effective active area for high HER reaction kinetics over LSmZO catalyst, which might be originated from the plenty of active sites. Furthermore, these results are in consistent with determined value surface coverage (*Г*) and diffusion coefficient (*D*).

#### Hydrogen evolution studies

**Fig. 8:**
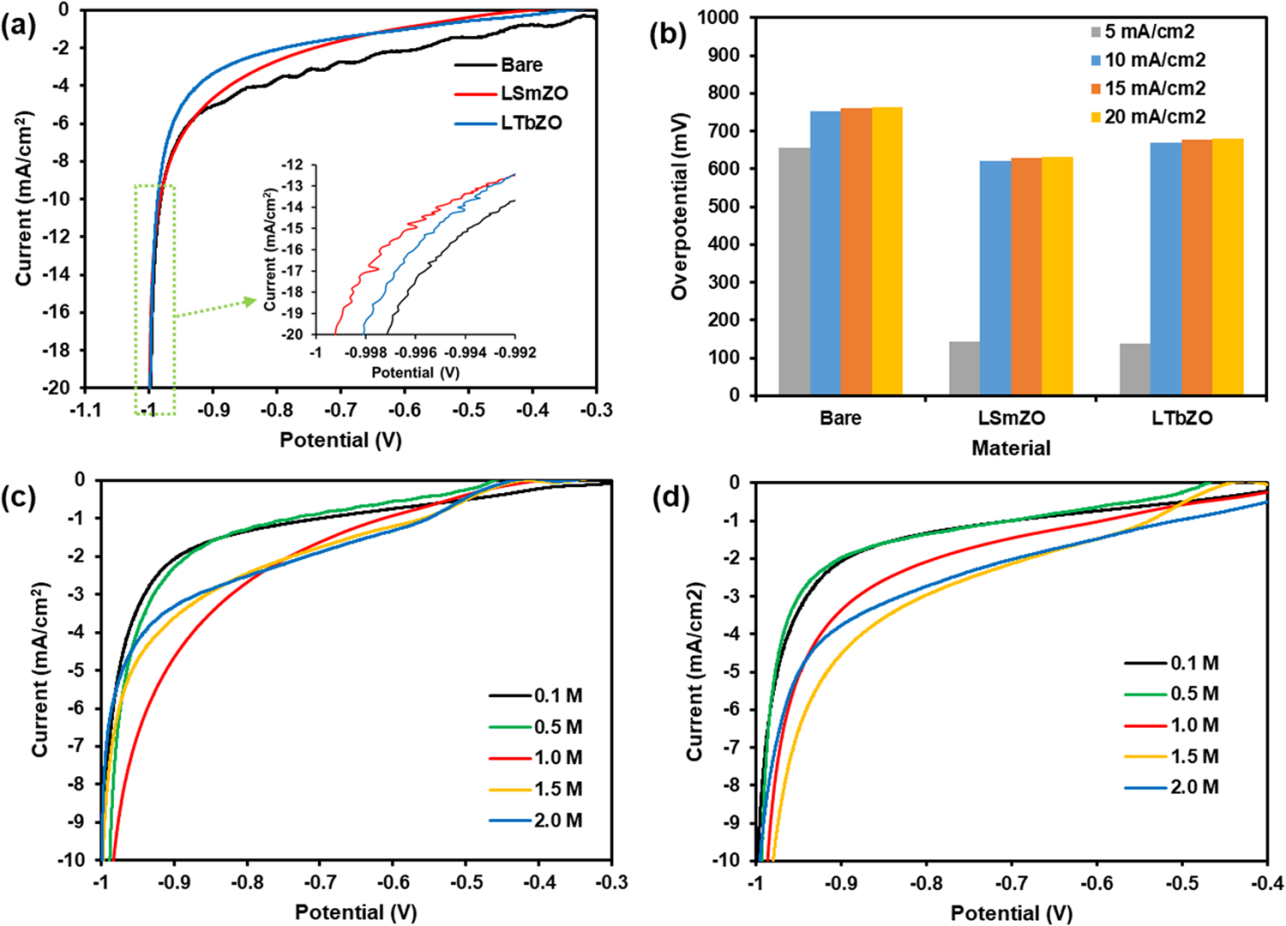
Electrochemical hydrogen production performance tests: *a* LSV curves of bare, LSmZO and LTbZO in 1 M KOH electrolyte at a scan rate of 10 mV·s^−1^ and its corresponding overpotentials (**b**) at 5, 10, 15 and 20 mA·cm^−2^; **c** and **d** LSV curves of LSmZO and LTbZO in different concentrations of KOH electrolyte (0.1–2 M) at a scan rate of 10 mV·s^−1^, respectively

The characteristics of bare, LSmZO and LTbZO electrocatalysts were explored in 1.0 M KOH alkaline medium @ 10 mV·s^−1^ scan rate. In Fig. 8a, surprisingly bare electrode displays uppermost HER activity, particularly at low current densities. Among the synthesized materials, LSmZO considerably outperforms the LTbZO delivering the lowest potential 0.998 V at a current density of 20 mA·cm^−2^. However, different trends were observed from the Tafel plots (Fig. 8b). As shown in Fig. 8b, a low overpotential *ŋ*_10_ = 502 mV is observed in 1.0 M KOH in LSmZO compared to overpotential *ŋ*_10_ = 750 mV of bare, and the LTbZO shows even lower overpotential *ŋ*_10_ = 435 mV at the same concentration of electrolyte. Figure 8c and d show LSV curves of the HER activity of LZO, LSmZO and LTbZO in several KOH concentrations (2.0 M), and the results are summarized in Table 3. The nanostructured LSmZO and LTbZO resulted in similar HER performance to deliver current density of 10 mA·cm^−2^. Both synthesized perovskite materials have comparable trends with HER activity increasing as the pH/KOH concentration increased from 0.01 (pH 12) to 1.0 M (pH 13.8), and then the HER performance slightly decreased in 2.0 M (pH 14.2) KOH. The results are comparable to those reported in the literature (Faid et al. 2022) [50]. Taking into account the microstructure changes in terms of adsorption/desorption of hydrogen on the surface of material, we have performed XRD measurements after the cycling stability test (Fig. 1b). Based on the obtained results, the peak intensity decreases with observation of extra peaks after the HER measurements. This situation can be assigned to the lattice distortion caused by the expansion/contraction during electrochemical hydrogen absorption/desorption (Monama et al. 2018).

**Table 3:** Comparative study of the HER performance of this work catalyst and reported electrocatalysts

Material	Electrolyte		*ŋ* (mV vs. RHE) at 10 mA·cm^−2^	-*b* (mV·dec^−1^)	*α*	*i*_0_ (µA·cm^−2^)	*TOF* (× 10^−4^ mol *H*_2_/s)	Reference
Bare	KOH (M)	1.00	750	138.7	0.581	0.001	0.982	This work
LZO	KOH (M)	1.00	502	255.2	0.772	427.7	2.622	This work
LSmZO	KOH (M)	1.00	502	123.6	0.530	1.166	0.440	This work
LTbZO	KOH (M)	1.00	435	102.6	0.433	0.068	0.454	This work
ZrO_2_ nanoparticles	NaOH (M)	1.00	443	139	-	-	-	Jaffri et al. (2023)
Zr_0.8_Ni_0.2_B_2_	KOH (M)	1.00	420	101.0	-	-	-	Jaffri et al. (2023)
Sr_2_ Fe_1.5_ Mo_0.5_ O_6− δ_	KOH (M)	1.00	375	172.0	-	-	-	Gao et al. (2021)
Ba_0.5_ Sr_0.5_ (Co_0.8_ Fe_0.2_)_1−_ _X_ P_x_ O_3− δ_	KOH (M)	0.1	370.0	138.4	-	-	-	Zhang et al. (2020)
Ba_0.5_ Sr_0.5_ (Co_0.8_ Fe_0.2_)_1−_ _X_ P_x_ O_3− δ_	KOH (M)	1.0	333.0	73.3	-	-	-	Zhang et al. (2020)
LaCoO_3_	KOH (M)	0.1	410.4	125.0	-	-	− 0.74	Wu et al. (2023)
Nd_1− x_ Co_x_ FeO_3_	KOH (M)	1.0	239.0	68.0	-	-	-	Ilanchezhiyan et al. (2021a, b)
SrTiO_3_	KOH (M)	0.1	399.4	179.0	-	-	-	Mohamed et al. (2022)
SrCo_0.70_ Fe_0.30_ O_3−δ_	KOH (M)	1.0	332.0	189.3	-	-	-	Zhang et al. (2019)
PrBaCo_2_O_5 + δ_	KOH (M)	0.1	429.0	183.1	-	-	-	Sun et al. (2019)
Pr_0.5_ (Ba_0.5_ Sr_0.5_)_0.5_ Co_0.8_ Fe_0.2_ O_3–δ_	KOH (M)	1.0	237.0	45.0	-	-	-	Xu et al. (2016)
BaSrCoMoO_6_	KOH (M)	1.0	325.0	142.0	-	-	-	Karki et al. (2022)
Ba_0.5_Sr_0.5_ Co_0.8_ Fe_0.2_ O_3−δ_	KOH (M)	1.0	470.0	174.0	-	-	-	Karki et al. (2022)
SmFeO_3_	KOH (M)	1.0	312.0	78	-	-	-	Ilanchezhiyan et al. (2021a, b)
SmFe_1−x_ Er_x_ O_3_	KOH (M)	1.0	298.0	55	-	-	-	Ilanchezhiyan et al. (2021a, b)

**Fig. 9:**
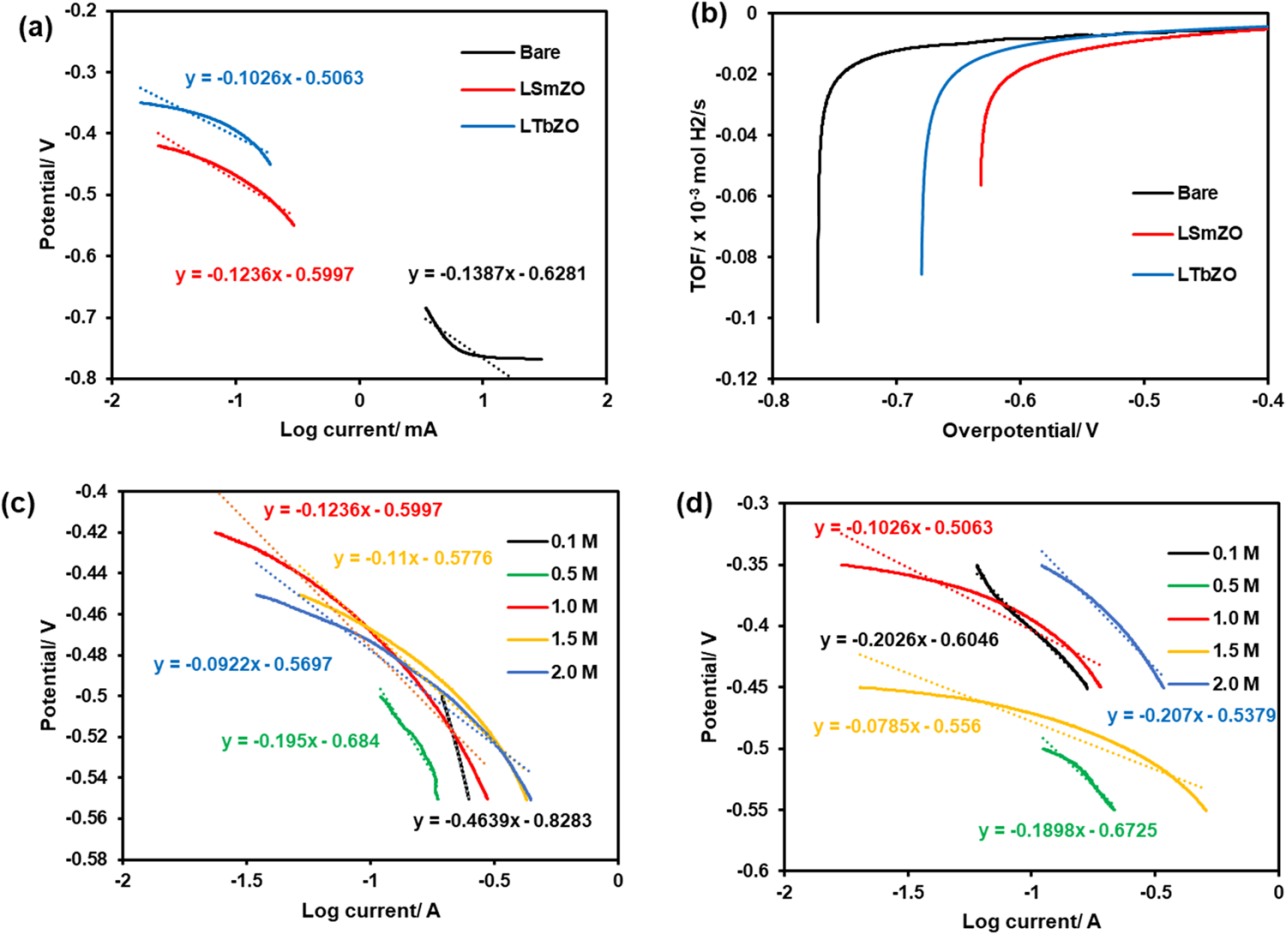
Tafel plot in 1 M KOH (**a**) and *TOF* as a function of overpotential curves of bare (**b**), LSmZO and LTbZO in 1 M KOH electrolyte at a scan rate of 10 mV/s; **c** and **d** Tafel plots of LSmZO and LTbZO in different concentrations of KOH electrolyte (0.1–2.0 M) at a scan rate of 10 mV/s, respectively

The electrochemical HER activity of the synthesized material was studied by Tafel plots. Tafel plots are useful tools employed to assess the rate-limiting step of the HER. Figure 9(a) displays the Tafel plots of bare, LSmZO and LTbZO, which are derived from the LSV at 1.0 M KOH. As shown in Fig. 9(a, c and d), the Tafel polarization curves and relationship between overpotential (*ŋ*) and current density (log *j*) are demonstrated (Shinagawa et al. 2015; He et al. 2017).

The corresponding Tafel slope values were 255.2 mV·dec^−1^ for LZO parent material, 123.6 mV·dec^−1^ for LSmZO and 102.6 mV·dec^−1^ for LTbZO, which suggests that hydrogen evolution followed the Volmer–Heyrovsky mechanism (Swathi et al. 2022; Hussain et al. 2021). The low Tafel slope value suggests faster HER kinetics, whereas lager Tafel slope indicates a slower HER kinetics (Swathi et al. 2022). From the obtained results, LTbZO seems to have better HER performance, with Tafel slope of 102.6 mV·dec^−1^ which suggests faster HER kinetics.

The turnover frequency (*TOF*) was used to assess the intrinsic HER activity of the bare, LSmZO and LTbZO electrocatalysts. The *TOF* values at similar concentrations of 1.0 M KOH are calculated, as shown in Fig. 9(b). The bare exhibit much larger *TOF* than LSmZO and LTbZO. In particular; the *TOF* of bare is 0.98 10^−4^ s^−1^ at an overpotential of 750 mV, more than 2 times as high as that of LSmZO and LTbZO catalysts. The synthesized catalyst recorded *TOF* values of 0.44 × 10^−4^ s^−1^, 0.45 × 10^−4^ s^−1^ at an overpotential of 502 mV, 435 mV for LSmZO and LTbZO, respectively. As it has been reported in literature, the improved HER activity due to the presence of electrocatalyst is likely to originate from the enhanced water adsorption and dissociation process (Zhao et al. 2023). Figure 9c and d display Tafel slopes of nanostructured perovskite LSmZO and LTbZO in (0.1–2.0) M KOH concentrations. Tafel slopes of around 120 mV·dec^−1^ are obtained for LTbZO at all studied KOH concentrations excluding 2.0 M, indicating that HER is controlled by the first electron transfer step (Volmer step), which is governed by the blockade of the electrochemical water dissociation (Sarmad et al. 2022).

$$\text{Volmer step }\sim 120 \text{mV}/\text{dec}$$

$${\text{H}}_{2}\text{O}+\text{M}+{\text{e}}^{-}\to \text{M}-{\text{H}}_{\text{ads}}+{\text{OH}}^{-}$$

LSmZO displays Tafel slopes’ dependence on KOH concentration with Tafel slope of around 463.9, 195.0, 123.6, 110.0 and 92.2 mV·dec^−1^ in 0.1, 0.5, 1.0, 1.5 and 2.0 M KOH, respectively. Similar trends at lower concentration (0.1–1.0 M KOH) with Tafel slope of above 120 mV·dec^−1^ is observed with LSmZO, indicating volmer step as rate-determining step (rds) (Faid et al. 2022; Wang et al. 2019). Tafel slope of around 90 mV·dec^−1^ relates to rds of second electrochemical desorption Heyrovsky step (Wang et al. 2019). This observation implies that the HER mechanism for perovskite LSmZO changes with KOH concentration to Volmer-Heyrovsky mechanism.

$$\text{Heyrovsky step},\text{ electrochemical desorption}\sim 40\text{mV}/\text{dec}$$

$${\text{H}}_{2}\text{O}+\text{M}-{\text{H}}_{\text{ads}}+{\text{e}}^{-}\to {\text{H}}_{2}+{\text{OH}}^{-}$$

**Fig. 10:**
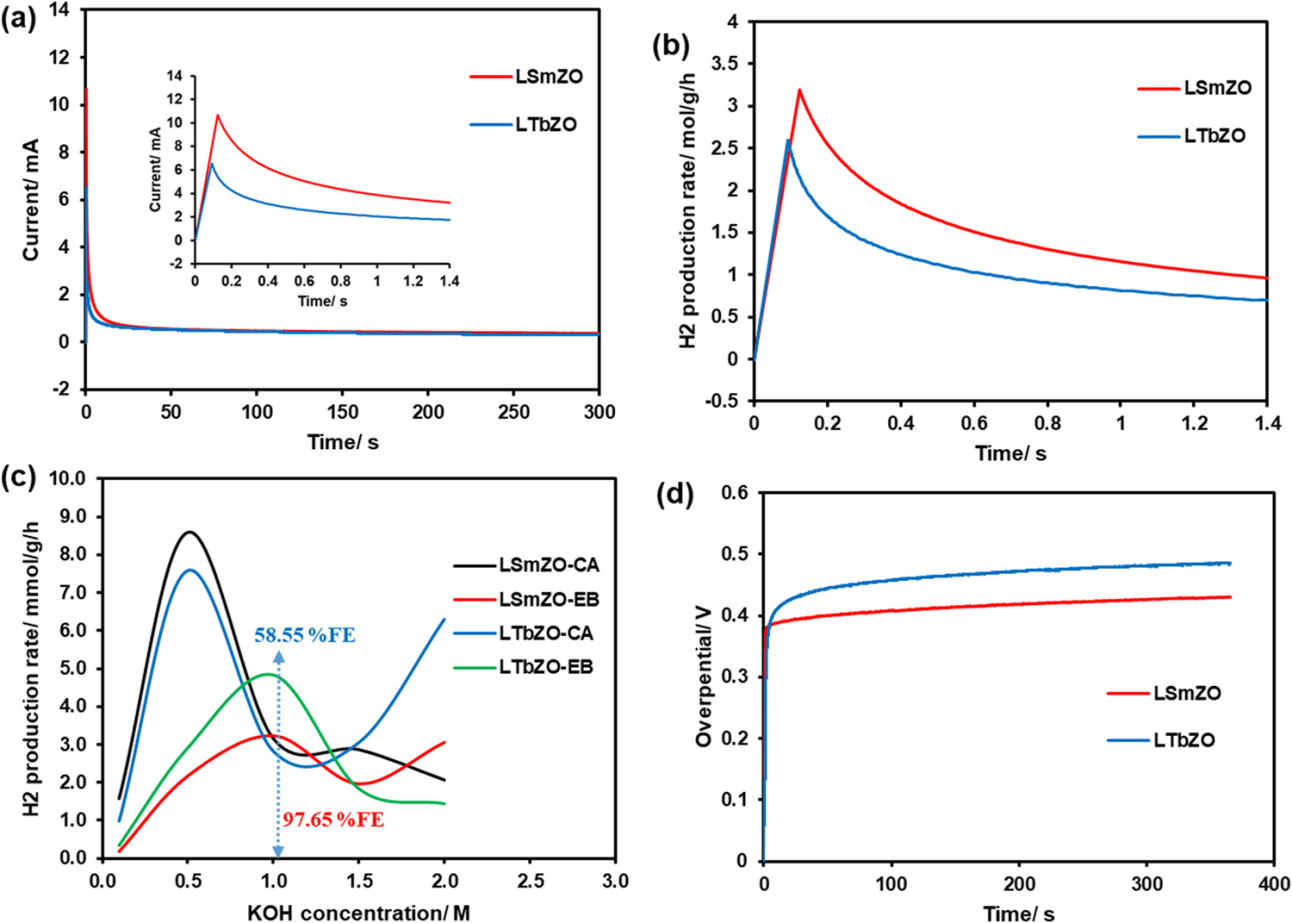
**a** *CA* curve of LSmZO and LTbZO (inset: enlargement graph). **b** The hydrogen production rate using Faraday law on the *CA* data. **c** Hydrogen production rate determined from the *CA* data and electronic balance (*EB*) as a function of the electrolyte concentration (0.1–2.0 M), and the Faradaic efficiency (*FE*) at 1.0 M KOH. **d** CP curve of LSmZO and LTbZO at 10 mA/cm^2^ for 3600 s

In order to evaluate the stability of the catalysts, aging experiments at controlled potentials were carried out. Figure 10(a) shows the chronoamperometry test measured in 1.0 M KOH when the applied potential is 0.3 V (vs. Ag/AgCl) for all the catalysts synthesized. It can be seen that the LSmZO catalyst has an increase of only 11 mA, demonstrating higher robustness of LSmZO than LTbZO. These results are agreeing with a small band gap of LTbZO determined from XPS which suggest less stability. The amount of the produced hydrogen was calculated by integration of areas under chronoamperometric curves obtained at each potential and applying Faraday’s law (Khaligh et al. 2021; Bottini et al. 2017). Hence, the amount of hydrogen produced (H_2_) per hour per gram of catalyst is determined using Eq. (1) (Khaligh et al. 2021).

1 $${H}_{2}=\frac{3600 \times \text{ I}}{nFm}$$

The representative parameter I is a current from CA measurement; *m* is the amount of catalyst (g) used; *n* for 2 electrons required to produce 1 mol of hydrogen, and *F* is a Faraday’s constant. Figure 10b shows that the amount of hydrogen generated with LTbZO was 2.52 mmol/g/h at optimum condition, which was slightly lesser than the LSmZO (3.34 mmol/g/h) catalyst, and similar approach to calculate *H*_2_ molecule produced was reported (Bottini et al. 2017). Faradaic efficiency was also determined at the potential of 0.3 V (Fig. 10c). The LTbZO showed a Faradaic efficiency of only 58.55%, while LSmZO showed higher % Faradaic efficiencies of 97.65%. The results indicated that LTbZO and LSmZO catalysts were comparable to benchmark Pt/C and Ru@MWCNT electrodes with %FE of 46.99 and 85.88 at 1.5 V, respectively (Kweon et al. 2020). Additionally, electrochemical stability of LTbZO and LSmZO was evaluated in basic electrolyte, by performing the chronopotentiometry test on the synthesized electrocatalyst (Fig. 10d). The overpotential of both electrocatalysts (LTbZO and LSmZO) gradually increased with time at a current density of 10 mA·cm^−2^. The overpotential increase was about 0.25 mV and 0.34 mV for LSmZO and LTbZO, respectively. This increase is correlated to degeneration in performance of electrocatalyst because of partial shedding on the electrode material and as a result hinders the effective contact of the catalysts with the electrolyte due to generated *H*_2_ (Wang et al. 2023).

### Electrochemical impedance studies

**Fig. 11:**
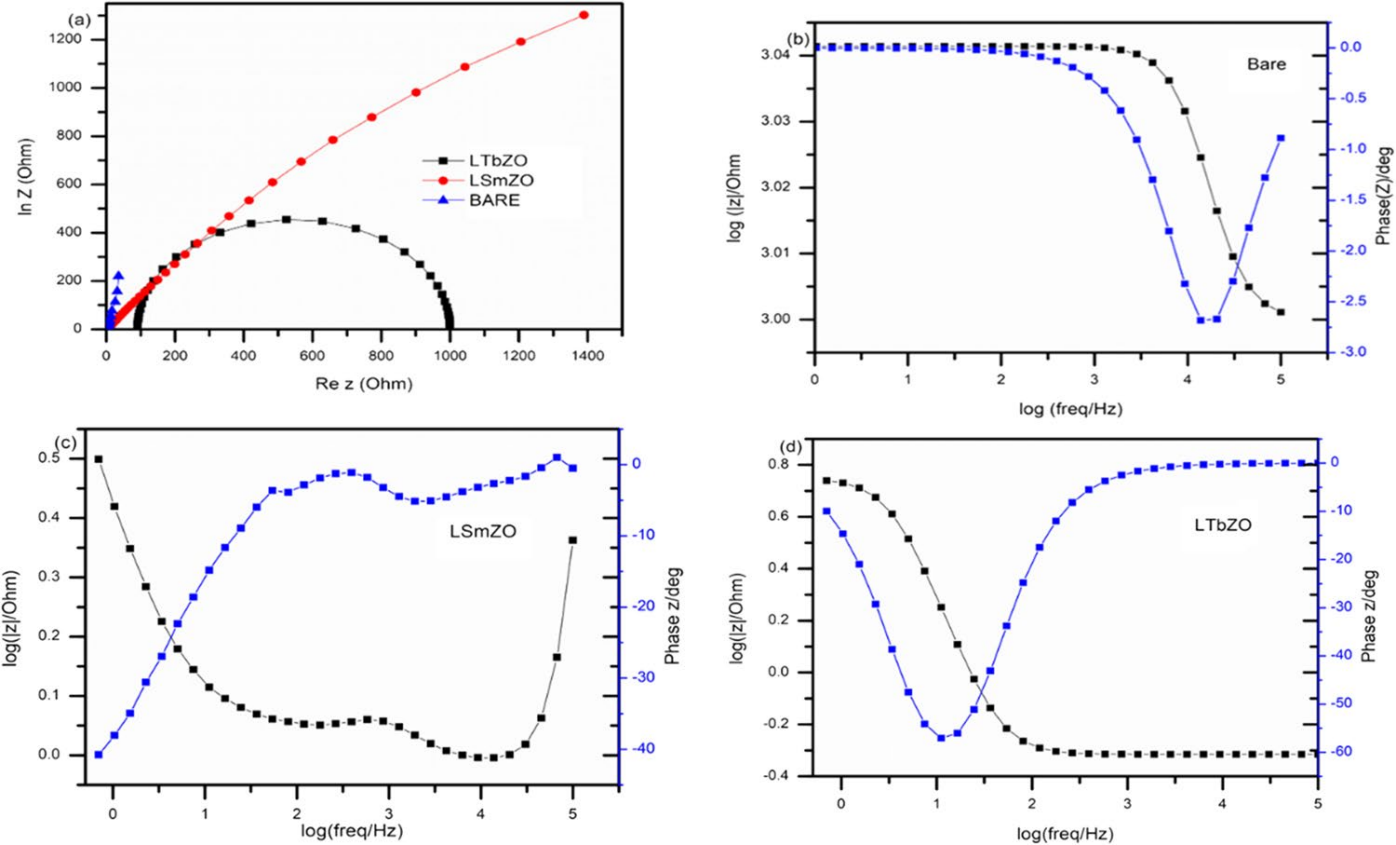
**a** *EIS* for all materials’ frequency range of 10 kHz to 0.1 Hz and amplitude of 5 mV in 1 M KOH, Bode plot of bare (**b**), LSmZO (**c**) and LTbZO (**d**)

The customary three-electrode system of *EIS* measurements were done in order to assess the kinetics of charge transfer in these samples. The diameter of the semicircle is considered to be related to charge transfer at the electrode/electrolyte interface of the HER. A smaller diameter resembles to more efficient charge transfer resistance (*R*_*ct*_) and better conductivity. Figure 11(a) shows the Nyquist plots of bare, LTbZO and LSmZO electrodes, respectively, which are collected by scanning from 10 kHz to 0.1 Hz. LTbZO material had a smaller semicircle diameter compared to bare and LSmZO materials, indicating more efficient charge transfer. The *R*_*ct*_ values were 0.5453 Ω, 1.481 Ω, 6.293 Ω with Faradaic capacitance of 7.319 × 10^−3^, 3.47 × 10^−3^, 0.223 × 10^−3^ for LTbZO, LSmZO and bare, respectively. The efficient charge transfer resistance of LTbZO could be attributed to high *ECSA* than LSmZO which resulted in better conductivity. The Bode plots of LSmZO and LTBZO are shown in Fig. 11b, c and d and are used to estimate the lifetime of electrons for the materials. The lifetime (*τ*) of the synthesized compounds was determined using Eq. (2), where (*f*_max_) is its maximum peak frequency (Ramaripa et al. 2023).

2 $$\tau =\frac{1}{2\uppi ({f}_{max})}$$

The estimated electron lifetime of the electrodes was found to be that bare 53 ms, 62 ms, and 88 ms for bare, LSmZO and LTbZO, respectively. The long electron lifetime of the catalyst could be correlated to better conductivity of the material.

## Conclusion

This work successfully synthesised novel perovskite materials based on LSmZO together with its counterpart material, LTbZO, through a sol–gel reaction and was entirely characterized. In a basic condition, we have demonstrated that doping Sm^3+^ and Tb^3+^ into the A-site of perovskite material exhibits a highly active catalyst for HER. In an alkaline medium, the effects of LSmZO and LTbZO loading on Ni foam substrates were investigated electrochemically. The observed promotional roles of introducing lanthanides (M^3+^) on perovskite catalyst resulted in improved HER activity as part of hydrogen production with enhanced *TOF* values. LTbZO perovskite, compared to LSmZO, displayed meaningfully improved catalytic activity with a smaller Tafel slope. Moreover, the Tafel parameters, the *b* and *α* values, indicated that the HER rate-determining step was the Volmer mechanism or the Volmer mechanism together with one of the other two reactions. The improved HER performance may be due to the increased oxygen vacancy brought by Tb/Sm-doping, together with an enhanced *ECSA* and faster electron transfer. It was discovered that LSmZO’s HER electrocatalytic activity produced 3.344 mmol/g/h of hydrogen (Faradaic efficiency of 97.65%). To the best of our knowledge, this is the first time LTbZO and LSmZO perovskite oxides have been reported for efficient hydrogen evolution electrocatalysis under basic conditions, which we believe could open the door for more perovskite materials for HER.

## Data Availability

Data and materials are available on request.
